# The Black Box warning: Exploring the ethical considerations and mitigating medicolegal risk in prescribing antidepressants to young adults

**DOI:** 10.1192/j.eurpsy.2025.460

**Published:** 2025-08-26

**Authors:** P. Thiagayson

**Affiliations:** Institute of Mental Health, Singapore, Singapore

## Abstract

**Introduction:**

Since October 2004, antidepressants have had a black-box warning indicating that they are associated with an increased risk of suicidal thinking, feeling, and behavior in children and adolescents. On 2nd May 2007, the United States Food and Drug Administration ordered that all antidepressant medications carry an expanded black-box warning stating an increased risk of suicidal symptoms in young adults aged 18 – 24.

The initiation of antidepressants in the young adult population present unique patient safety considerations and medicolegal risks for physicians. Unlike the triadic doctor-parent-patient relationship in a child or adolescent patient, the doctor-patient realtionship in a young adult is a dyadic one. Hence, enlisting the help of a trusted adult to supervise the young person who has been newly initiated on antidepressants is more challenging.

**Objectives:**

In this poster, we explore the unique ethical considerations in initiating antidepressants in the young adult population and the steps clinicians may take to mitigate their medicolegal risk in treating this population. The ethical considerations of autonomy, beneficence and non-maleficence are analysed. The measures an individual clinician may take in clinical decision-making and follow-up to mitigate medicolegal risk are also discussed.

**Methods:**

A literature search was conducted to determine the clinical considerations and prescription patterns in prescribing antidepressants to young adults. The legislation surrounding medication prescription in major jurisdictions were explored. Current literature on medicolegal risk management was studied to come up with recommendations on mitigating medicolegal risk when initiating antidepresants in young adults.

**Results:**

Initiating antidepressants in a young adult is a collaboratively undertaken medical decision. A thorough evaluation is required to determine if antidepressant initiation is warranted. Young adults initiated on antidepressants must be closely monitored for increased suicidality. The clinician should offer to psychoeducate a member of the young adult’s support network on his diagnosis and treatment, and apprise this person of the black box warning. . If there are imminent grave risks to the young person or others, confidentiality may have to be broken. Clear detailed documentation of the clinical considerations and discussion with the young person is essential.

**Conclusions:**

Initiating antidepressants in young adults presents clinicians with unique patient safety and medicoleagl risk concerns. Steps in clinical decision-making and follow-up may be undertaken by clinicians to mitigate this risk.

**Disclosure of Interest:**

None Declared

